# Administrative burden in Swiss nursing homes and its association with care workers’ outcomes—a multicenter cross-sectional study

**DOI:** 10.1186/s12877-023-04022-w

**Published:** 2023-06-02

**Authors:** Dietmar Ausserhofer, Waltraud Tappeiner, Heike Wieser, Christine Serdaly, Michael Simon, Franziska Zúñiga, Lauriane Favez

**Affiliations:** 1 Claudiana Research, College of Health Care-Professions, Bolzano-Bozen, Italy; 2 Institute of Nursing Science, Department of Public Health, University of Basel, Bernoullistr. 28, 4056 Basel, Switzerland; 3 Serdaly&Ankers Snc, 210 Route de Florissant, 1231 Conches, Switzerland; 4 School of Engineering and Management Vaud, HES-SO University of Applied Sciences and Arts Western Switzerland, Yverdon-les-Bains, Switzerland

**Keywords:** Nursing homes, Nursing care, Administrative tasks, Administrative burden, Burnout, Professional, Job dissatisfaction, Intention to leave

## Abstract

**Background:**

Care workers in nursing homes often perform tasks that are rather related to organizational or management activities than ‘direct patient care’. ‘Indirect care activities’, such as documentation or other administrative tasks are often considered by care workers as a burden, as they increase overall workload and keep them away from caring for residents. So far, there is little investigation into what kind of administrative tasks are being performed in nursing homes, by which type of care workers, and to which extent, nor how administrative burden is associated with care workers’ outcomes.

**Purpose:**

The objective of this study was to describe care workers’ administrative burden in Swiss nursing homes and to explore the association with four care worker outcomes (i.e., job dissatisfaction, emotional exhaustion, intention to leave the current job and the profession).

**Methods:**

This multicenter cross-sectional study used survey data from the Swiss Nursing Homes Human Resources Project 2018. It included a convenience sample of 118 nursing homes and 2′207 care workers (i.e., registered nurses, licensed practical nurses) from Switzerland’s German- and French-speaking regions. Care workers completed questionnaires assessing the administrative tasks and burden, staffing and resource adequacy, leadership ability, implicit rationing of nursing care and care worker characteristics and outcomes. For the analysis, we applied generalized linear mixed models, including individual-level nurse survey data and data on unit and facility characteristics.

**Results:**

Overall, 73.9% (*n* = 1′561) of care workers felt strongly or rather strongly burdened, with one third (36.6%, *n* = 787) reporting to spend 2 h or more during a "normal" day performing administrative tasks. Ratings for administrative burden ranged from 42.6% (*n* = 884; ordering supplies and managing stocks) to 75.3% (*n* = 1′621; filling out the resident’s health record). One out of four care workers (25.5%, *n* = 561) intended to leave the profession, whereby care workers reporting higher administrative task burden (OR = 1.24; 95%CI: 1.02–1.50) were more likely to intend to leave the profession.

**Conclusion:**

This study provides first insights on care workers’ administrative burden in nursing homes. By limiting care workers’ burdensome administrative tasks and/or shifting such tasks from higher to lower educated care workers or administrative personnel when appropriate, nursing home managers could reduce care workers’ workload and improve their job satisfaction and retention in the profession.

## Background

Nurse shortage in long-term care settings is a global challenge and is expected to worsen in the upcoming years considering the increase of older care-dependent persons in need of institutional care [1]. As the high care workload is often managed with low staffing levels, it might create a ‘vicious circle’, as the imbalances leading to higher personnel turnover contribute to an increasing workload for those care workers remaining. Internationally, turnover rates, i.e., the percentage of care workers leaving nursing homes, range between 19 and 55% [2]. Turnover rates are higher in nursing homes in comparison to other care settings [3]. For instance, a longitudinal study in Switzerland reported that 28% of care workers in nursing homes had already changed their job 5–6 years after completing their education compared to 17% of hospital care workers [4].

High turnover rates represent a huge problem for nursing home managers, as ensuring adequate and stable staffing levels is essential to provide person-centered care for residents and to prevent adverse events [3, 5]. Turnover is often the consequence of negative care worker outcomes, such as job dissatisfaction, emotional exhaustion and the intention to leave the job or even the profession [2, 6]. In a recent study, French et al. explored working conditions and nurse outcomes among 33′462 registered nurses in hospitals (*n* = 29′859) and nursing homes (*n* = 3′603) in the U.S., just before the outbreak of the Covid-19 pandemic [7]. Among registered nurses in nursing homes, 44% felt burned out, 28% were not satisfied with their current job, and 30% intended to leave their employer within the next year. Research has linked individual factors (e.g., care workers’ age, health status, affective organizational commitment), work environment (e.g., leadership and support, physical and psychological workload, time pressure and work stress, self-determination/autonomy, provision of person-centered care) and organizational factors (e.g., nursing home size) to these negative care worker outcomes [2, 8–12].

Care workers often complain about performing activities that they consider as bureaucratic and administrative in nature, which might be an additional factor explaining above-mentioned negative care worker outcomes. Compared to what has recently been described as “fundamental nursing care”, i.e., care activities related to hygiene, nutrition, elimination, mobility [13], these tasks are often perceived by care workers as not being a part of clinical nursing. As such, ‘administrative and indirect care activities’ can be considered by care workers as burdensome, hence often receiving lower priority and being left undone [14, 15]. Jackson, Anderson and Maben described nursing work as a conglomerate of physical, emotional, cognitive and organizational labour, where nurses often have to consider health care system demands simultaneously with the specific care demands of their patients [16]. Yet, few studies have aimed to understand organizational labour and nurses’ administrative tasks in nursing homes and hospitals. One of the few is Michel, Waelli, Allen and Minivielle, who explored the content and meaning of nurses’ administrative work in acute and long-term care settings in a comparative case study [17]. Administrative tasks were grouped into the following six domains: documenting patient records, coordinating activities and exams/interventions, managing patient flow, transmitting information, monitoring and reporting quality indicators and ordering supplies and stock management. Grosso et al. described in their qualitative study in the acute-care setting administrative tasks as “being out of the nursing role” that should be done by secretaries or other personnel [18]. In a following cross-sectional study, nearly all nurses (*n* = 693, 94.5%) reported to perform at least one administrative task during their last shift [19]. Nurses indicated the following reasons for performing administrative tasks: “compensating the lack of resources”, “being pressed by the organizational culture”, and “dealing with unexpected clinical events”. In a recent Swiss study applying a time and motion analysis of nursing activities, Michel, Grarcia Manjon, Pasquier and Ortoleva Bucher observed that registered nurses on an internal medicine unit spent 97 min per 12,5 h dayshift (i.e., 12.3%) and auxiliary staff 173 min per 12,5 h-shift (i.e., 23.1%) on activities, such as housekeeping, transportation and transfer of patients, and logistic tasks [20]. Similarly, Qian, Yu and Hailey observed that care workers in two Australian nursing homes spent 17.6% of their working time on indirect care activities, including administrative tasks [21]. In summary, these studies demonstrate that nurses often spend a considerable amount of their working time performing administrative and bureaucratic tasks. Yet, a common understanding and definition of “administrative tasks”, as well as which administrative tasks belong to nursing care or not, is lacking. For the purpose of our study, we considered as “administrative and indirect care activities” those six domains that have been identified and described in the qualitative study from Michel et al. [17].

Little is known about care worker’s administrative tasks in acute and long-term care settings. To our knowledge, no study explored the specific issue of administrative tasks done by care workers in nursing homes and the burden involved. Care workers’ administrative burden in nursing homes might be another factor explaining job dissatisfaction, emotional exhaustion and intention to leave the current job and/or the profession. It is important to understand what administrative activities care workers perform in nursing homes, to what extent they are perceived as burdensome and if they are related to negative care workers’ outcomes. Such evidence is necessary to develop strategies to retain care workers in nursing homes and prevent turnover. The objective of this study was therefore to describe care workers’ administrative burden in Swiss nursing homes and to explore the association with four care worker outcomes (i.e., job dissatisfaction, emotional exhaustion, intention to leave the current job and the profession), adjusting for facility, unit and work environment factors.

## Methods

### Study design

This was a multicenter cross-sectional study using survey data from the Swiss Nursing Homes Human Resources Project (SHURP) 2018.

### Sample and setting

A convenience sample of 118 nursing homes (NHs) in Switzerland's German- and French-speaking regions with 2′207 care workers, i.e., registered nurses (3 years of education at Swiss Universities of Applied Sciences or cantonal nursing schools) and licensed practical nurses (2–3 years education at vocational schools), working at least 20% were included in this analysis. The mean response rate to the care worker survey was 66%, ranging from 12.7% to 98.2% at facility level. As previously reported [22], NHs participating in this study were (1) invited NHs that had participated in the first edition of the SHURP study (2013–2015) [23] and accepted to participate in this new edition, (2) randomly selected NH from all NHs fulfilling the inclusion criteria in the German- and French-speaking part of Switzerland and invited to participate, or (3) NHs that were willing to participate and contacted the study team directly without being invited. Inclusion criteria were that each NH was recognized as such by cantonal authorities and had a minimum of 20 beds.

### Data collection

The survey was administered, as appropriate, in two language versions, German and French, between September 2018 and October 2019. All directors of the participating NHs gave written consent to participate in the study. For care workers, sending back the voluntary care worker questionnaire in the pre-franked envelope was considered informed consent.

### Variables and measures

We assessed four *care worker outcomes*, i.e., job dissatisfaction, emotional exhaustion, intention to leave the current job and intention to leave the profession. We used single items to measure overall job dissatisfaction (i.e., 4-point Likert-type scale ranging from ‘very unsatisfied’ to ‘very satisfied’), emotional exhaustion (i.e., single item from the Maslach Burnout Inventory [24] with a 7-point Likert-type scale ranging from ‘never’ to ‘daily’) and intention to leave the profession (i.e., 5-point Likert-type agreement scale ranging from ‘strongly disagree’ to ‘strongly agree’) as previously used in hospital [25] and NH studies [23]. To measure intention to leave the current job we used three items from the Michigan Organizational Assessment Questionnaire [26, 27], each using a 5-point Likert-type agreement scale ranging from 0 to 4, with higher numbers indicating stronger agreement [9]. The scale was calculated as sum over all items and dichotomized into ‘intention to leave (0)’ vs. ‘no intention to leave (1–12)’.

The main explanatory variable was *care workers’ administrative burden*, i.e., the self-assessed burden of performing six specific administrative and indirect care activities (4-point Likert-type scale ranging from ‘weak’ to ‘strong’), as previously described in qualitative studies [17, 18]: (1) filling out resident’s health record, (2) coordinating activities, exams or appointments for residents, (3) managing/administrating residents' admissions and discharges, (4) exchanging information (orally or written) with colleagues or within the interprofessional team, (5) evaluating residents with assessment instruments (e.g., Resident Assessment Instrument), and (6) ordering supplies and managing stocks. The original items were translated from English to German and French and adapted to the nursing home setting (e.g., ‘residents’ instead of ‘patients’). The explanatory factor analysis of the German and French versions’ internal structure of the six administrative tasks revealed a good fit for both language versions, suggesting a one-dimensional solution, i.e., Tucker Lewis Index of factoring reliability = 0.93 (German = 0.93, French = 0.85), Root Mean Square Error of Approximation index = 0.09 (German = 0.09, French = 0.07) and the 90% Confidence Interval: 0.08–0.11 (German = 0.08–0.10, French = 0.09–0.17), Cronbach’s alpha = 0.83 (German = 0.84, French = 0.80). Therefore, we calculated the scale’s mean score and used it for further analyses. We also measured care workers’ overall self-reported administrative burden (single item using the same 4-point Likert-type) and the time spent performing administrative tasks on a normal working day (single item with the four answer options ‘less than 1 h’, ‘between 1 and 2 h’, ‘between 2–3 h’ and ‘more than 3 h’).

All potential confounding and control variables, including facility and unit characteristics, perceptions of work environment factors (i.e., staffing and resources adequacy, leadership, working overtime), implicit rationing of nursing care and care worker characteristics, are described in Table 1. Except for care workers’ administrative burden, instruments and items had already undergone validity and reliability testing [23]. We report evidence on the internal structure (i.e., Cronbach’s alpha) of all instruments used in this study in Table 1.

**Table 1:** Study variables and measurements

Variable name	Description	Measurement
***Outcome variables***		
Job dissatisfaction	Single self-developed item assessing care workers’ satisfaction with their job [23, 28]	4-point Likert-type scale ranging from 1 ‘very satisfied’ to 4 ‘very unsatisfied’ dichotomized into ‘satisfied (1–2)’ vs. ‘dissatisfied (3–4)’
Emotional exhaustion	Single item from the Maslach Burnout Inventory assessing how often care workers felt emotionally exhausted from their work [24]	7-point Likert-type scale ranging from ‘never’ to ‘daily’ dichotomized into ‘few times a month or less often (0–4)’ vs. ‘once a week or more often (5–7)’
Intention to leave the current job	3 items assessing care workers’ turnover intention (“currently looking for another job”, “often thinking about quitting current job”, “probably looking for a job next year”) [9, 26, 27]	Sum over the three items each rated on a 5-point Likert-type agreement scale ranging from ‘strongly disagree (0)’ to ‘strongly agree (4)’; summarized over the three items and dichotomized into ‘no intention (0)’ vs. ‘intention to leave the current job (1–12)’; Cronbach’s α (in this study) = 0.89
Intention to leave the profession	Single self-developed item assessing if care workers intended to leave the profession and to take a job outside nursing [23, 28]	5-point Likert-type agreement scale ranging from ‘strongly disagree’ (0) to ‘strongly agree’ (4) dichotomized into ‘no intention (0–3)’ vs. ‘intention to leave the profession (4–5)’
***Explanatory variables***		
Care workers’ administrative burden	Mean score of the 6-items on care worker’s burden related to six administrative tasks [17, 18]	4-point Likert-type scale ranging from 1 ‘weak’ to 4 ‘strong’; Cronbach’s α (in this study) = 0.83
***Control variables***		
Facility characteristics		
Language region	Nursing homes in the German- or French-speaking region	1 = German-speaking 2 = French-speaking
Nursing home size	The facility's size, based on the number of long-term beds	1 = Small (< 50 beds) 2 = Medium (50–100 beds) 3 = Large (> 100 beds)
Ownership status	The type of nursing home based on financing	1 = Public 2 = Private subsidized 3 = Private
Unit characteristics		
Full-time equivalent per 100 beds	Full-time equivalent positions divided by number of beds, multiplied by 100	Number
Skill mix level	Percentage of all full-time equivalents per unit who are registered nurses	Number
Work environment		
Leadership	5-item “Nurse manager ability, leadership, and support of care workers” subscale of the Practice Environment Scale–Nursing Work Index, assessing direct supervisors in terms of the support they provided, their competency, back-up in decision-making, praise and recognition given, and the use of mistakes as learning opportunities rather than criticisms [29]	4-point Likert-type scale from 1 ‘strongly disagree’ to 4 ‘strongly agree’; Scale built with mean over items; Cronbach’s α (in this study) = 0.86
Staffing and resources adequacy	3-item subscale “Staffing and resources adequacy” of the Practice Environment Scale–Nursing Work Index, assessing whether there was enough time and opportunity to discuss resident care problems, enough qualified personnel to provide quality resident care, and enough staff to get the work done [29]	4-point Likert-type scale ranging from 1 ‘strongly disagree’ to 4 ‘strongly agree’; Scale built with mean over items; Cronbach’s α (in this study) = 0.75
Implicit rationing of nursing care	Mean score of the 21-item Basel Extent of Rationing of Nursing Care-Nursing Home version [30]	5-point Likert-type scale with the following response options: 0 ‘activity was not necessary’, 1 ‘never’, 2 ‘seldom’, 3 ‘sometimes’, or 4 ‘often’; Cronbach’s α (in this study) = 0.93
Care workers’ socio-demographic/professional characteristics		
Gender	Care worker gender	1 = Female 2 = Male
Age	Care worker age in years	Years (in six categories)
Educational background	Care worker professional education	1 = Registered nurse 2 = Licensed practical nurse
Tenure in current facility	Care worker tenure in current facility	Years (in three categories)
Employment level	Care worker employment level	% of employment (20–100), grouped in three categories for reporting
Main shift	Shift care worker most often works	1 = Regular change of shifts 2 = Day or evening shift 3 = Night shift
Working overtime	Frequency care worker works more than 30 min overtime	1 = Almost every shift 2 = Once a week 3 = Less frequently

### Data analyses

Descriptive statistics (i.e., frequencies, percentages, means, standard deviations) were calculated to describe the variables measured. We used Fisher’s Exact test to explore differences between care workers’ professional backgrounds with regard to the administrative burden. To explore the relationship between care workers’ administrative burden and the four care worker outcomes, 2-level binomial generalized linear mixed models were used to account for the clustering of care worker data within facilities. We first computed the Intraclass Correlation 1 (ICC1) to assess the variability at the facility level of the outcome and explanatory variables. Based on the ICC1, which was above the threshold of 0.05 for the main explanatory (i.e., administrative burden) and control variables (i.e., work environment), multilevel modeling was applied [31].

For each of the four care worker outcomes we report unadjusted (‘crude’) associations and two adjusted models: 1) administrative burden and control variables without staffing and resources adequacy, leadership and rationing of nursing care; and 2) administrative burden, control variables and staffing and resources adequacy, leadership and implicit rationing of nursing care. To compare the models' relative fits, we used Akaike’s information criterion; a lower value indicates a better fit. Data analyses were performed with R (version 4.0.3, R Core Team, 2017) using the rptR package for the calculation of ICC1 [32] and the lme4 package for binomial generalized linear mixed models [33]. In the data for unit and facility characteristics we had no missing values. In the care worker survey data missing values varied between 0.0% (i.e., educational background, employment level) and 2.4% (i.e., tenure in current facility). A P-value of less than 0.05 was considered significant.

## Results

### Sample description

We used a sample of 2′207 care workers, 88.2% of which were female. More than two-thirds were over 30 years old (70.9%), had less than 10 years of professional experience in their current NH (74.5%) and worked part-time (71.0%) Almost half worked with regular changes of shifts (49.8%) and worked overtime for more than 30 min less than once a week (49.7%). Of the 108 Swiss NHs included in the study, most NHs were medium-sized (between 50 and 100 beds) and private or privately-subsidized. Table 2 summarizes care worker, unit and facility characteristics.

**Table 2:** Facility, unit, and care worker characteristics

**Characteristics**			**Missing (%)**
**Facility characteristics (*n* = 118 facilities), %—n**			
Nursing home size			0.0
Small (20–49 beds)	22.9	27	
Medium (50–99 beds)	47.4	56	
Large (≥ 100)	29.7	35	
Ownership status			0.0
Public	45.8	54	
Private subsidized or private	54.2	64	
Language region			0.0
German-speaking	83.1	98	
French-speaking	16.9	20	
Service area			0.0
Urban	72.0	85	
Rural	8.5	10	
Agglomeration	19.5	23	
**Unit characteristics (*n* = 368 units), Median – Interquartile range**			
Bed capacity,	24	11	0.0
Full-time equivalent per 100 beds (FTE/100 beds)	49.3	22.3	0.0
Skill mix level (% registered nurse)	28.8	16.5	0.0
**Care worker characteristics (*n* = 2′207 care workers), %—n**			
Gender (female)	88.9	1´947	0.8
Age (years)			0.3
< 21	6.2	137	
21–30	22.9	505	
31–40	19.5	428	
41–50	19.9	439	
51–60	25.4	557	
> 60	6.1	135	
Educational background			0.0
Registered nurse (3–4 years of education)	48.9	1´079	
Licensed practical nurse (3 years of education)	51.1	1´128	
Tenure in current facility			2.4
0–4 years	53.6	1´154	
5–9 years	20.9	451	
≥ 10 years	25.5	549	
Employment level			0.0
< 51%	14.5	320	
51%-90%	56.5	1´247	
91%-100%	29.0	640	
Main shift			0.1
Regular change of shifts	49.8	1´099	
Day or evening shift	41.4	912	
Night shift	8.8	194	
Working overtime			0.3
Almost every shift	7.0	153	
Once a week	43.2	950	
Less frequently	49.8	1´097	

### Variable result description

#### Care workers’ burden and differences between registered nurses and licensed practical nurses

Overall, 73.9% of care workers rated the administrative burden as strong or rather strong, with 36.6% spending 2 h or more during a "normal" day performing administrative tasks (see Table 3). The percentage of respondents rating the burden of respective administrative tasks surveyed as strong or rather strong ranged from 75.3% (“filling out the resident’s health record”) to 42.6% (“ordering supplies and managing stocks”). As summarized in Table 3, we observed significant differences regarding self-reported burden of administrative tasks between registered nurses' and licensed practical nurses, except for “ordering supplies and managing stocks”. Compared with licensed practical nurses, registered nurses reported higher overall administrative burden for five out of six administrative tasks, as well as regarding spending more than 2 h on a "normal" day performing administrative tasks.

**Table 3:** Care workers’ administrative burden by educational level (*N* = 2´207)

Administrative burden items	Total (*N* = 2′207), % (n)	Registered nurses (*n* = 1′079), % (n)	Licensed practical nurses (*n* = 1´128), % (n)	*P* value^c^
Filling out the resident’s health record.^a^	75.3 (1´621)	79.4 (842)	71.2 (779)	< *0.001*
Coordinating activities, exams or appointments.^a^	49.5 (1´045)	52.8 (550)	46.4 (495)	*0.003*
Managing/administrating residents’ admissions and discharges.^a^	55.5 (1´159)	61.3 (637)	49.8 (522)	< *0.001*
Exchange of information (orally or written to colleagues, within the interprofessional team).^a^	52.3 (1´117)	54.6 (575)	50.2 (542)	*0.046*
Evaluating residents with the assessment instruments (with RAI, BESA or PLAISIR).^a^	73.0 (1´510)	76.7 (798)	69.2 (712)	< *0.001*
Ordering supplies and managing stocks.^a^	42.6 (884)	42.5 (438)	42.7 (446)	0.93
Overall administrative burden.^a^	73.9 (1´561)	80.4 (837)	67.6 (724)	< *0.001*
Spending 2 h or more during a "normal" day performing administrative tasks.^b^	36.6 (787)	48.2 (507)	25.5 (280)	< *0.001*

^a^Percentage agreement (strong or rather strong)

^b^Percentage agreement (2 h or more)

^c^Fisher’s Exact test, *P* < .05 highlighted in italic

#### Care worker outcomes, work environment and rationing of nursing care

As Table 4 shows, a considerable amount of care workers reported job dissatisfaction (17.9%) and emotional exhaustion (18.8%). From the respondents, 70.1% had at least some intention to leave their current job and one out of four care workers intended to leave the profession (25.5%). The mean administrative burden was rated as rather strong (mean: 2.64). Care workers rated adequate staffing and resources at the neutral midpoint (mean: 2.66) and strongly felt they were supported by leadership (mean: 3.18). The mean rating for implicit rationing of nursing care documentation was 0.91 (seldom). While ICC1 for administrative burden, work environment and rationing of nursing care were substantial ranging between 0.066 and 0.201, for three out of four nurse outcomes (i.e., job dissatisfaction, emotional exhaustion and intention to leave the profession) the ICCs were < 0.05, indicating no substantial variation between facilities (see Table 4).

**Tab 4:** Characteristics of variables under study (*N* = 2´207)

Variables	% (n)	Mean (SD)	Facility level, ICC1 (95%CI)
Nurse outcomes			
Job dissatisfaction^a^	17.9 (383)	-	0.018 (0–0.044)
Emotional exhaustion^b^	18.8 (414)	-	0.031 (0.001–0.068)
Intention to leave the current job^c^	70.1 (1′536)		0.071 (0.025–0.103)
Intention to leave the profession^d^	25.5 (561)	-	0.027 (0.005–0.057)
Administrative burden	-	2.64 (0.63)	0.066 (0.037–0.096)
Work environment			
Leadership	-	3.14 (0.63)	0.135 (0.092–0.181)
Staffing and resources adequacy	-	2.66 (0.67)	0.201 (0.145–0.257)
Implicit rationing of nursing care	-	0.91 (0.59)	0.151 (0.105–0.199)

*CI* Confidence interval, *SD* Standard deviation, *ICC* Intraclass correlation

^a^Percentage agreement (‘very satisfied (1)’ or ‘satisfied’ (2))

^b^Percentage agreement (once a week or more often)

^c^Percentage agreement (1–12 points on the sum score ranging from 0 to 12)

^d^Percentage agreement (‘agree’ (4) or ‘strongly agree’ (5))

### Association between administrative burden and care workers’ outcomes

In all models adjusted for facility, unit, and care worker characteristics, administrative burden was significantly associated with care workers’ outcomes (see Table 5): Higher care workers’ administrative burden was associated with higher job dissatisfaction (Odds Ratio (OR): 1.32 [95%-CI: 1.09–1.61]), higher emotional exhaustion (OR: 1.66 [95%-CI: 1.36–2.03]), higher intention to leave the current job (OR: 1.42 [95%-CI: 1.21–1.66]) and higher intention to leave the profession (OR: 1.52 [95%-CI: 1.28–1.81]) (models 1).

**Table 5:** Administrative burden regressed on care workers’ outcomes, along with work environment, implicit rationing of nursing care, facility, unit and care worker characteristics (*N* = 2´207)

**Care workers’ outcomes**	**Crude models** ^a^			**Multiple adjusted model 1 (without staffing and resources adequacy, leadership and rationing of nursing care)**^a,b^			**Multiple adjusted model 2 (with staffing and resources adequacy, leadership and rationing of nursing care)**^a,b^		
	**OR**	**95%CI**	**SE**	**OR**	**95%CI**	**SE**	**OR**	**95%CI**	**SE**
**Job dissatisfaction**									
Administrative burden	1.47***	1.22–1.76	0.09	1.32**	1.09–1.61	0.10	0.96	0.76–1.20	0.11
Leadership	0.21*	0.17–0.26	0.10				0.30***	0.24–0.38	0.12
Staffing and resources adequacy	0.27*	0.23–0.33	0.10				0.60***	0.46–0.77	0.13
Implicit rationing of nursing care	2.99*	2.47–3.63	0.10				1.53***	1.19–1.96	0.13
**Emotional exhaustion**									
Administrative burden	1.84***	1.53–2.21	0.09	1.66***	1.36–2.03	0.10	1.22	0.98–1.52	
Leadership	0.39***	0.32–0.46	0.08				0.56***	0.45–0.69	
Staffing and resources adequacy	0.34***	0.29–0.41	0.09				0.71**	0.56–0.90	
Implicit rationing of nursing care	3.76***	3.10–4.56	0.10				2.38***	1.87–3.04	
**Intention to leave the current job**									
Administrative burden	1.46***	1.26–1.68	0.07	1.42***	1.21–1.66	0.08	1.07	0.89–1.28	0.09
Leadership	0.21***	0.17–0.25	0.09				0.30***	0.24–0.37	0.11
Staffing and resources adequacy	0.28***	0.24–0.33	0.08				0.59***	0.48–0.73	0.11
Implicit rationing of nursing care	3.34***	2.81–3.97	0.09				1.75***	1.40–2.18	0.11
**Intention to leave the profession**									
Administrative burden	1.49***	1.27–1.75	0.08	1.52***	1.28–1.81	0.08	1.24*	1.02–1.50	0.10
Leadership	0.43***	0.37–0.50	0.08				0.59***	0.48–0.71	0.10
Staffing and resources adequacy	0.42***	0.36–0.49	0.08				0.66***	0.53–0.82	0.11
Implicit rationing of nursing care	2.31***	1.94–2.74	0.09				1.49***	1.19–1.85	0.11

*OR* Odds ratio, *SE* Standard error, *CI* Confidence interval

^a^Generalized linear mixed model (binomial) with facility level as random effect

^b^Adjustments for facility (i.e., language region, nursing home size, ownership status), unit (i.e., full-time equivalent per 100 beds, skill mix level) and care worker (i.e., gender, age, educational background, tenure in current facility, employment level, main shift, working overtime) characteristics

*P*-value: *** < 0.001, ** < 0.01, * < 0.05

In model 2, controlled for staffing and resources adequacy, leadership and rationing of nursing care, care workers reporting higher administrative burden had still higher odds for reporting intention to leave the current profession (OR: 1.24 [95%-CI: 1.02–1.50]). Care worker-perceived staffing and resources adequacy was the strongest and most consistent factor explaining care worker outcomes. Higher ratings for staffing and resources adequacy and leadership were associated with less care workers reporting job dissatisfaction, emotional exhaustion, intention to leave the current job and the profession, while higher levels of implicit rationing of nursing care were associated with more negative care worker outcomes.

## Discussion

With this study we described care workers’ self-assessed burden of performing administrative and indirect care activities in Swiss nursing homes and explored the association with four care worker outcomes (i.e., job dissatisfaction, emotional exhaustion, intention to leave the current job and the profession). Almost three out of four care workers felt strongly or rather strongly burdened. One out of four care workers intended to leave the profession, whereby care workers reporting higher administrative burden were more likely intending to leave the profession.

In our study, one third of care workers and nearly half of registered nurses reported to spend 2 h or more during a "normal" day performing administrative tasks. Qian, Yu, Zhang, Hailey, Davy and Nelson reported similar results in two nursing homes with care workers spending 18% of their work time on indirect care activities [34]. As our data show, registered nurses reported higher administrative burden than licensed practical nurses, which might be associated with the higher amount of time registered nurses spend on performing administrative and indirect care activities. Nursing home management should carefully analyze which administrative work needs to be done by which type of care worker according to their scope of practice, e.g., to streamline registered nurses’ administrative tasks and/or shifting such tasks from higher to lower educated care workers or administrative personnel when appropriate. This would not only help reduce administrative burden but might as well release time that registered nurses can spend on direct care activities, which is known to be associated with better nursing home resident outcomes [35, 36].

The most burdensome administrative task reported by our sample of care workers was filling out residents’ health record. While documentation is an essential part of nursing care, it is a rather time-consuming activity, as the qualitative study from Olivares Bøgeskov and Grimshaw-Aagaard [37] on the experiences of registered nurses and nurse leaders in a hospital setting confirmed. We hypothesize that the burden of filling out residents’ health records might partly be associated with inefficient IT-systems and/or technological support. In our study, almost all Swiss nursing homes had already implemented electronic health records and nursing documentation systems. However, this does not necessarily mean that these systems support care workers to perform documentation tasks in an effective and efficient way. In a previous analysis of the same sample we found that Swiss nursing home care workers perceived the electronic health record systems as useful, yet only half of the care workers reported sufficient computers on their unit to allow timely documentation [22]. An adequate and flexible IT-system is crucial to organize the work time of care workers in a way that it reduces the burden associated with performing such tasks. Care workers’ opportunity to develop or update nursing care plans or document nursing care in real time at the patient’s bedside via mobile devices (e.g., tablets or smartphones) may reduce the burden of performing these administrative activities.

Another highly burdensome administrative task was evaluating residents with the assessment instruments (e.g., Resident Assessment Instrument). Although this is a clinical activity that is intended to support care planning and clinical decision making, it is perceived by nurses as a rather administrative task [17]. Due to the competencies needed to perform it, “evaluating residents with the assessment instruments” is a registered nurse or licensed practical nurse task, while administrative tasks like “ordering supplies and managing stocks” can be delegated to less qualified staff. Several administrative tasks (e.g., clinical assessment, care planning and documentation) are considered an integral part of nursing work. However, nurses’ organizational tasks related to information flow and management of patients across the healthcare system are often unnoticed and unrecognized, not only by health care organizations, but also by nurses themselves [16]. Allen pointed out that these are crucial tasks and should not be seen by nurses as taking them away from patients (and from their “clinical” work) [38]. Further research is needed to explore more in depth where care workers see an added value for the residents and their work in performing administrative tasks related to the nursing process, including clinical assessment, and where it unnecessarily adds to their burden as information and data are mainly collected for administrative purposes (e.g., insurance companies, monitoring of care activities).

We found a considerable amount of care workers feeling emotionally exhausted, dissatisfied with their current job and with the intention to leave their job and even their profession. As our analyses revealed, higher administrative burden was statistically significantly associated with worse nurse outcomes (multiple adjusted model 1). After further adjusting for staffing and resources adequacy, leadership and rationing of nursing care, higher administrative burden remained only an independent significant predictor for higher intention to leave the profession (multiple adjusted model 2). On one hand, our finding that administrative burden lost its effect for the three other nurse outcomes is somehow not surprising. It is well known from the scientific literature that staffing levels and leadership, as well as implicit rationing of nursing care are key factors determining both resident [39–42] and care worker outcomes [8, 9]. Thus, these factors might play a more important role than the administrative burden and moderate its effect, since higher administrative burden might lead to rationing of necessary nursing care and to the perception of lacking staffing resources to comply with all the tasks at hand. Similarly Bratt and Gautun in a Norwegian survey study found that working conditions (i.e., physical and mental workload and time pressure) were the strongest predictors for wishing to leave the current job in the nursing home [10]. On the other hand, administrative burden did remain stable in explaining care workers’ intention to leave the profession in our Swiss nursing home sample. From the hospital setting it is known that worse work environment, including leadership, interprofessional collaboration as well as nurses’ individual factors, such as older age, female gender and working full-time are associated with higher intention to leave the profession [43]. In line with Aloisio, Coughlin and Squires self-determining and having more autonomy on decisions pertaining to performing administrative tasks (e.g., what to do and what not, what to delegate, what do to differently) could be a strategy to help care workers reduce their administrative burden [8]. Since this is the first study investigating administrative burden in nursing homes and its relationship with care workers’ outcomes, further investigation is necessary to gain a deeper understanding on the role of burdensome administrative tasks in care workers’ retainment, job satisfaction or turnover intentions and decisions.

Scientific work in this field has just begun and “administrative work” within or without of what is considered as “fundamental nursing care” [13], being necessary or unnecessary, is still not well defined. Consequently, within a holistic understanding of nursing, it remains unclear, if and to what extent the burden associated with performing administrative tasks might be avoidable or not, respectively acceptable, or not. Several concepts like “indirect care”, “non-nursing tasks”, “organizational work” or “miscellaneous activities” have been investigated in previous studies aiming to differentiate between activities that are within and out of nurses’ scope of practice [18–20, 38, 44]. While a clear conceptual definition would be helpful to better understand care workers’ burden with administrative work in nursing homes and other settings, this seems to be a challenging endeavor. As Michel et al. found, there are differences in the type and extent of administrative work performed by nurses in acute and long-term care settings, and consequently how much burden care workers experience in performing these tasks [17]. These findings are not surprising, as there are obvious differences between healthcare settings in the provision of nursing care, e.g., due to patient or resident care needs, or the work environment (i.e., technological support, staffing and skill mix levels). Moy et al. suggest that future studies should measure care workers’ administrative burden, e.g., related to documentation, across different settings, as it is not clear which tasks are especially felt as burdensome and by whom, and with which magnitude [45]. To measure “administrative burden” in our study we developed a tool based on qualitative research from Michel et al. revealing six nurses’ administrative tasks: “documenting the patient record”, “coordination of activities and examinations or appointments”, “management of admissions and discharges of residents”, “transmission of information (verbal and written)”, “resident assessment”, and “ordering supplies and stock management” [17]. Although there might be more specific administrative tasks being part of nursing in different care settings, the measurement applied in our study consisting of care workers’ self-reports on the burden with these general administrative tasks could contribute to the measurement and comparison of care workers’ administrative burden across different settings. However, further work on theoretical and conceptual underpinnings of administrative burden and its’ measurement are necessary.

### Strengths and limitations

The strengths of our study are the large sample of Swiss nursing homes and care workers and high response rates. This allowed us to describe the administrative burden and explore the relationship with care workers outcomes in nursing homes. The measurement instrument we developed was based on empirical work and showed good first validity and reliability. There are several limitations that need to be considered. Given the cross-sectional nature of our study, findings cannot be interpreted for causality or the temporal direction of the found associations. For instance, nurses who intend to leave the profession might experience overall high work-related burden and therefore be more likely to report higher administrative burden. Moreover, our findings might not be generalizable to other health care settings. Our findings might be affected by common method bias as care workers’ administrative burden and nurse outcomes were assessed within the same survey. Two of our primary outcomes were care workers’ intention to leave. It remains unclear if intention actually leads to the decision to leave the nursing home or the profession and if the associations found can be confirmed with actual turnover rates.

## Conclusions

Our study revealed that a high number of care workers in Swiss nursing homes felt burdened with performing administrative tasks. After adjusting for major work environment factors and implicit rationing of nursing care, administrative burden was still associated with care workers’ intention to leave the profession. These first insights into care workers’ administrative burden in nursing homes can inform the development of interventions to reduce the workload related to ‘indirect care activities’ and to improve care workers’ job satisfaction and retention in the profession. Further research is needed on the conceptualization and measurement of administrative burden, differentiating between tasks that are inherently linked to nursing and those that are not as well as exploring options to reduce administrative burden.

## Data Availability

The datasets generated and analyzed during the current study are available from the corresponding author on reasonable request.
